# The transition in the ventral stream from feature to real-world entity representations

**DOI:** 10.3389/fpsyg.2014.00695

**Published:** 2014-07-02

**Authors:** Guy A. Orban, Qi Zhu, Wim Vanduffel

**Affiliations:** ^1^Department of Neuroscience, University of ParmaParma, Italy; ^2^Laboratorium voor Neuro-en Psychofysiologie, Department of NeuroscienceKU Leuven, Leuven, Belgium

**Keywords:** 3D shape, retinotopy, actions, 2D shape, material properties

## Abstract

We propose that the ventral visual pathway of human and non-human primates is organized into three levels: (1) ventral retinotopic cortex including what is known as TEO in the monkey but corresponds to V4A and PITd/v, and the phPIT cluster in humans, (2) area TE in the monkey and its homolog LOC and neighboring fusiform regions, and more speculatively, (3) TGv in the monkey and its possible human equivalent, the temporal pole. We attribute to these levels the visual representations of features, partial real-world entities (RWEs), and known, complete RWEs, respectively. Furthermore, we propose that the middle level, TE and its homolog, is organized into three parallel substreams, lower bank STS, dorsal convexity of TE, and ventral convexity of TE, as are their corresponding human regions. These presumably process shape in depth, 2D shape and material properties, respectively, to construct RWE representations.

## INTRODUCTION

This brief thought-provoking perspective paper complements the review devoted to the extrastriate neuronal properties published in Physiological reviews (Orban, 2008). At that time (Orban, 2008; Nassi and Callaway, 2009) the properties of infero-temporal neurons were not well understood, preventing a coherent picture of the function of monkey TE and its equivalent regions in man to be drawn. The present perspective paper attempts to correct this shortcoming. Since fMRI became available (Dubowitz et al., 1998; Logothetis et al., 1998; Stefanacci et al., 1998; Vanduffel et al., 1998) for systematic investigation in the alert monkey (Vanduffel et al., 2001), considerable progress has been made, through fMRI-guided monkey single-cell studies, and by parallel comparative imaging in humans and monkeys. In addition, the connections of TE cortex have recently been reassessed (Saleem et al., 2007, 2008; Ungerleider et al., 2008; Gerbella et al., 2010; Kravitz et al., 2013), allowing a tight comparison between anatomical connectivity and functionality.

## RETINOTOPIC ORGANIZATION OF THE VISUAL SYSTEM

**FIGURE 1:**
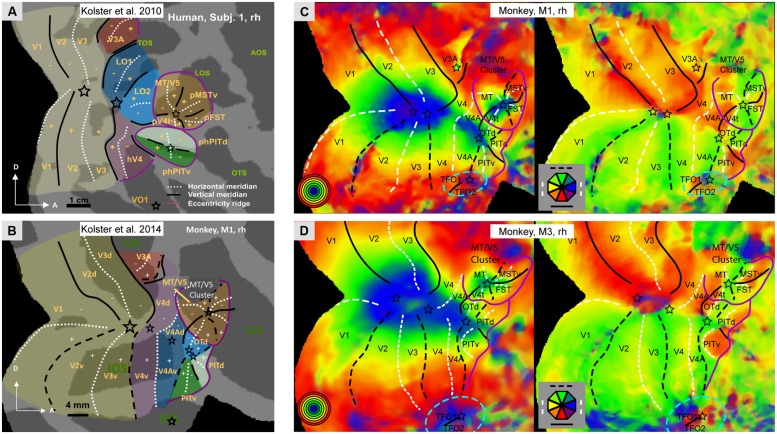
** (A,B)** Schematic representation of the retinotopic organization of occipital cortex: in humans (**A**, subject 1, rh) and in monkeys (**B**, monkey M1, rh); Modified from Kolster et al. (2014).**C,D**: Polar angle and eccentricity maps for monkeys M1 **(C)** and M3 **(D)**, same data as Janssens et al. (2014) but lower threshold. Black lines: vertical meridians (full: upper, dashed: lower), white dashed lines: horizontal meridians, stars: central visual field representation; purple lines: eccentricity ridges; In **A,B**: LuS: lunate sulcus, STS: superior temporal sulcus; OTS occipito-temporal sulcus; TOS: transverse occipital sulcus, LOS: lateral occipital sulcus, AOS: anterior occipital sulcus, OTS occipito-temporal sulcus; Other nomenclature: see Abbreviations. In **C,D** blue stippled elliptic outlines mark additional retinotopic regions (TFO1/2) ventral to V4A/PITv.

Our understanding of the retinotopic organization of the human visual system is largely due to fMRI. It is now established that human occipital cortex and neighboring parts of temporal and parietal cortex includes 15–17 distinct representations of the visual field. In addition to the three early visual areas V1-3, there is agreement (Wandell et al., 2007; Arcaro et al., 2009; Kolster et al., 2010) concerning hV4, LO1-2, the four areas of the MT cluster (MT, pMSTv, pFST, and pV4t), phPITd and phPITv (**Figure 1A**), and V6 (Pitzalis et al., 2006). There still is debate concerning the V3A complex which is subdivided into either two (V3A/B; Larsson and Heeger, 2006) or four areas (V3A/B/C/D; Georgieva et al., 2009). Dorsally, the V3A complex is bordered by V7 (Tootell et al., 1997), which is in fact the first parietal area, also designated IPS0 (Silver et al., 2005). Recently, V7 was reported to be part of a cluster of two areas, V7 (IPS0) and V7A (IPS1), sharing a central representation (Georgieva et al., 2009), a finding confirmed by using stereoscopically- instead of luminance-defined phase-encoded retinotopy stimuli (Kolster et al., 2011). This test also suggested that at more rostral levels the posterior parietal cortex (PPC) is retinotopically organized into 3–6 additional areas. Their complete characterization requires further work, since investigations thus far have relied mainly on polar angle analyses to define IPS2-5 (Silver and Kastner, 2009). On the other, ventral side of the occipital cortex Kolster et al. (2010) have described a single VO1 area (**Figure 1A**), although these data are also compatible with the presence of a second VO2 area, as described by Brewer et al. (2005). Finally, Arcaro et al. (2009) have shown that VO1-2 borders two additional retinotopic areas, PH1 and PH2, extending into the parahippocampal cortex. Thus in humans, a major difference exists between the dorsal and ventral visual pathways with respect to their retinotopic representation. The dorsal pathway retains a retinotopic organization, while the ventral pathway discards this organization beyond the phPIT cluster. It needs to be noted, however, that the most ventrally located occipito-temporal cortex processing scene information remains retinotopically organized. It has been suggested that at higher levels of the ventral pathway, eccentricity remains an important principle of organization (Levy et al., 2001), but this largely reflects the representation of large eccentricities in scene-processing regions.

The situation is very similar in the macaque. Its occipital cortex and neighboring parts of temporal and parietal cortex includes 14 retinotopic maps (**Figure 1B**): the three early areas V1-3, V4, and its two satellites (V4A and OTd), the two PITs (Janssens et al., 2014; Kolster et al., 2014), V3A, the four areas of the MT cluster (Kolster et al., 2009), and V6 in the parieto-occipital sulcus (Galletti et al., 1999). Cytoarchitectonic area TEO, which initially was proposed to contain a single retinotopic map (Boussaoud et al., 1991), in fact includes four different retinotopic maps: V4A, OTd, PITd, and PITv (Janssens et al., 2014; Kolster et al., 2014). It may be that neighboring cytoarchitectonic area TFO will undergo the same fate. Indeed, ventrally in occipital cortex, in front of the most peripheral part of V4 and below V4A, there is preliminary evidence (Janssens et al., 2014; Kolster et al., 2014) for another central representation, defining a cluster including two areas joined by that central representation. These areas have been tentatively labeled TFO1 and TFO2 (**Figures 1C,D**). The location in the dorsal bank of OTS and internal organization of this cluster suggest they may correspond to VO1-2 of humans. In humans VO1/2 are sensitive to color (Brewer et al., 2005) and color responses have been reported in a monkey PET study in a region that likely corresponds to TFO (Takechi et al., 1997). We propose that TFO1/2 are the starting point of the scene-processing pathway, consistent with recent fMRI activation and single cell recordings (Kornblith et al., 2013, but see Nasr et al., 2011). As in humans this pathway emphasizes the peripheral visual field (Kravitz et al., 2013). A number of parietal regions are retinotopically organized. Arcaro et al. (2011) described, in addition to DP, a pair of areas, CIP1 and CIP2, in the caudal part of the lateral bank of the IPS. In keeping with their location caudal to an extensive representation of peripheral visual field, CIP1/2 might be the monkey counterparts of the V7/V7A pair (Durand et al., 2009). This implies that human areas V3B-D have no counterpart in the monkey and are evolutionary novel areas. This is consistent with the caudal elongation of the IPS which in humans includes an occipital portion needed to bridge the enlargement of IPL (Grefkes and Fink, 2005). Further forward in monkey IPS, Arcaro et al. (2011) described a single hemifield representation, LIP, of which the central representation had been described by Fize et al. (2003).

**FIGURE 2:**
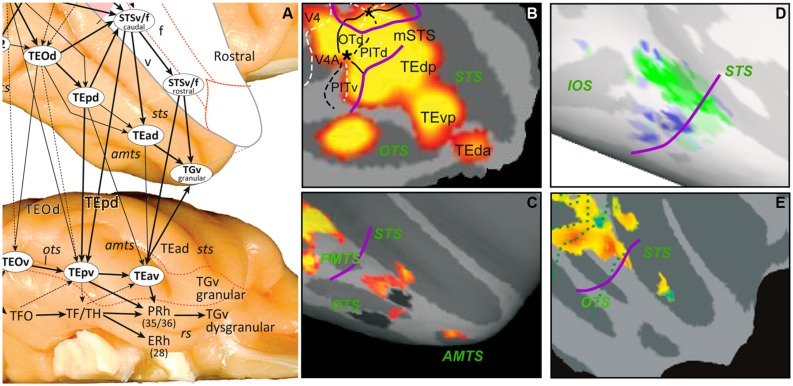
**(A)** The anatomical organization of monkey TE into three parallel substreams (from Kravitz et al., 2013); **(B–E)** SPMs showing activation sites in right IT for 2D shape, color, shape vs. no shape, and gloss. These were defined by the following subtractions: *intact* vs. *scrambled images* of objects **(B)**, *color* vs. *no color* mondrians **(C)**, *inact* vs. *scrambled images* of objects **(D)** main effect of *gloss*, independent of contrast **(E)**. In D the non-shape, selective voxels were strongly selective for material property, whereas shape-selective ones were not. Purple curved lines in **B–E**: approximate caudal boundary of TE. From Denys et al. (2004; **B**), Harada et al. (2009; **C**), Goda et al. (2014; **D**), and Okazawa et al. (2012; **E**).

In summary, the retinotopic organization of occipital cortex is remarkably similar in human and non-human primates, more than initially appreciated (Wandell et al., 2007). In addition, the organization beyond occipital cortex is also rather similar. The dorsal visual pathway of both humans and monkeys maintains a retinotopic organization, while the ventral pathway abandons this organization beyond TEO/the PIT monkey areas and their human homologs (phPITs). In both species the rostral limit of retinotopic cortex represents the peripheral visual field (purple lines in **Figure 1**). The most ventral, scene-processing pathway transiting through the parahippocampal cortex retains this organization at least in humans and possibly in monkeys (this ventral cortex is difficult to image in the monkey given the susceptibility artifacts, see Ku et al., 2011). Insofar as scene processing might be considered the qualitative counterpart of the metric processing of space in the dorsal pathway, the underlying principle may be that areas processing space, either quantitatively or qualitatively retain a crude retinotopic organization. In the monkey, the temporal cortex beyond TEO/the PITs includes mainly areas TE and TGv near the temporal pole (**Figure 2A**). In humans, LOC, which primarily corresponds to TE (Denys et al., 2004; Sawamura et al., 2005) is located several cm away from the temporal pole, suggesting that the TGv region has greatly expanded in humans. This raises the question by which functional organization principle, if any, the retinotopic organization has been replaced in these regions of temporal cortex.

## PITd PROCESSES 3D SHAPE FROM SHADING, ONE OF THE BUILDING BLOCKS OF SHAPE REPRESENTATION FOR REAL-WORLD ENTITIES

In monkeys, the fMRI study of Nelissen et al. (2009) indicates that the dorsal PIT is involved in processing 3D shape from shading. The fMRI activation of PITd corresponds to stronger neuronal responses for shading patterns reflecting 3D structure (Köteles et al., 2008). In humans, 3D shape from shading is similarly processed in a restricted occipito-temporal region (Georgieva et al., 2008). Matching the local maximum of this activation to a maximum-probability map of occipital retinotopic areas (Abdollahi et al., 2013) suggests that it is located near or in phPITd. In an effort to dissociate 3D shape from shading from simple flat luminance patterns, both Nelissen et al. (2009) and Georgieva et al. (2008) required joined activation in several specific contrasts for a region to be considered processing 3D shape from shading. Sereno et al. (2002) also reported 3D shape from shading responses in a somewhat broader region near PITd, including MT and FST in which several 3D shape cues, motion, shading, and texture converged. The importance of these observations derives from the fact that the image of any real-world object is necessarily (because of optics) characterized by two complementary components: a boundary that defines its 2D shape and a luminance pattern inside this boundary that defines its relief (shape in depth or 3D shape). These two complementary components depend in complex ways on the material properties and shape of the objects, as well as the direct and indirect light sources present in the scene. Nevertheless, 2D shape and 3D shape from shading combine to unambiguously define a visual representation of a real-world entity (RWE), whether an object, a plant, an animal, or a conspecific. RWE is preferred to the term *object* which is ambiguous, as the above listing shows. It is well established that boundary information is processed in V4 (Pasupathy and Connor, 2001) and is further elaborated in what is commonly called TEO (Brincat and Connor, 2004). Thus the most rostral retinotopic regions of the ventral pathway (**Figure 1B**), parts of cytoarchitectonic TEO, contain the elements required to generate visual representations of RWE. We propose that the primary function of TE, located beyond the retinotopic cortex, is to house the visual representations of RWEs, built by combining lower-level inputs from retinotopic cortex. The visual representation of RWEs can also be triggered by their images (Tanaka et al., 1991), and by even more simplified stimuli such as drawings (Denys et al., 2004).

The visual representations of RWEs are supposedly assembled in TE by combining inputs representing a boundary (or external contour) as well as elements of the luminance distribution inside that boundary. These internal elements can be either contours corresponding to extremes in the luminance distribution, or regions of constant or smoothly varying luminance. Indeed, this combinatorial view is supported by recent recordings in the ML face patch of the monkey, located just at the edge of retinotopic cortex. Almost all neurons in this patch are face selective (Tsao et al., 2006) and this selectivity arises from combining the geometry of the boundary with that of key internal features such as the eyes, nose, or mouth (Freiwald et al., 2009), but also includes the contrast levels in certain positions with respect to these features (Ohayon et al., 2012). However, this combination of 2D shape and 3D from shading does not exhaust the possible visual representations of RWEs, since the nature of RWEs is specified by not only their shape but also their material properties. Hence the representation of RWEs is build up from three main sources: features related to the 2D shape of the boundary in the image, and to the 3D shape, and material properties of the region enclosed by the boundary.

## REPRESENTATIONS OF REAL-WORLD ENTITIES IN TE

**FIGURE 3:**
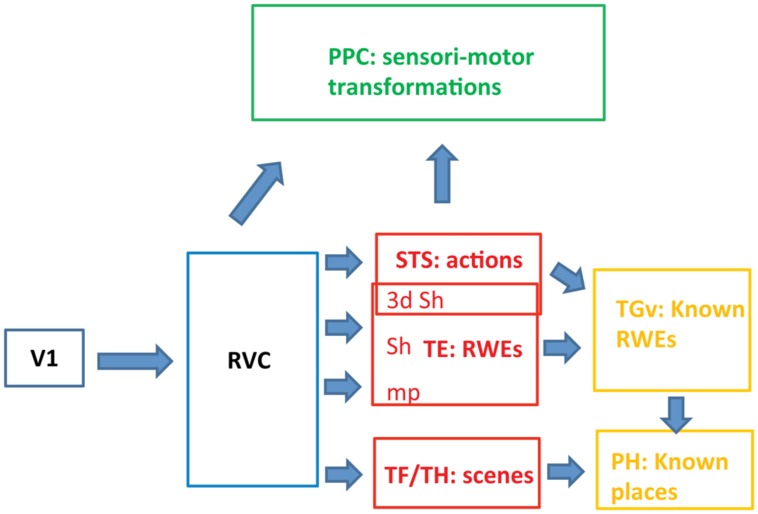
**Schematic view of the functional organization of the ventral pathway in the three levels (blue, red, and yellow).** RVC: retinotopic visual cortex includes the PITs, i.e., the posterior part of the IT complex; RWE: real world entity; sh: shape, mp: material properties, PH: parahippocampal cortex.

Recent anatomical data suggest that three parallel substreams operate within TE (**Figure 2A**), located in the lower bank of STS and in the dorsal and ventral parts of TE. We suggest that these three streams preferentially use features of 3D shape, 2D shape, and material properties, respectively, to build up RWE representations (**Figure 3**). This implies that functional segregation between these substreams is maximal at the transition between the retinotopic, feature level and the middle level (i.e., the TEO/TEp border in **Figures 2A,D**) and gradually blurs toward the rostral end of TE. Indeed, the three aspects defining RWEs (3D shape, 2D shape, and material properties) contribute in different proportions to the definition of given RWEs, and some cues belonging to one of the aspects may remain represented at more rostral levels, as for example color, one of the material cues (see below). According to this scheme the middle substream carries mainly 2D shape information, as evidenced by the subtraction *intact* minus *scrambled images* of objects, which mainly activates dorsal TE (**Figure 2B**; Denys et al., 2004; Sawamura et al., 2005; Lafer-Sousa and Conway, 2013). A long list of single-cell studies have been devoted to 2D shape selectivity in IT cortex (Logothetis and Sheinberg, 1996; Tanaka, 1996; Orban, 2008 for review), with some stressing the affine nature of the representation (Kayaert et al., 2005). This 2D shape substream also contains several face patches, such as the ML, and AL patches (Moeller et al., 2008).

The ventral TE substream may process material properties (for review see Fleming, 2014) which also contribute to the definition of RWEs (e.g., a tomato is red and smooth). This is supported by the color activation sites in ventral TE (**Figure 2C**; Harada et al., 2009; Lafer-Sousa and Conway, 2013). The other principal material property cue is texture (texture is also a cue for 3D shape; see Sereno et al., 2002; Orban, 2011). Little is known about texture processing in monkeys (see Köteles et al., 2008), but in humans ventral occipito-temporal cortex is heavily involved in texture processing (Peuskens et al., 2004; Cant and Goodale, 2007). Ku et al. (2011) have reported face patches in and around the ventral temporal cortex of the monkey: in ventral TE, area TF, entorhinal cortex, hippocampus, and region labeled ventral V4, which might have included TFO. Since the hairy monkey face and control stimuli (fruits, houses, and fractals) differed in texture, some of these activation sites (in particular the posterior ones) might actually reflect the texture differences rather than the presence of the face. Regions in PIT processing material properties have been investigated recently by Goda et al. (2014), who showed a clear segregation between shape and material properties at the level of PIT (**Figure 2D**), in agreement with our proposal. We propose that the third substream in the lower bank of STS processes 3D shape (Sereno et al., 2002; Yamane et al., 2008). This proposal is consistent with the presence in the lower bank of a small patch concerned with gloss (**Figure 2E**; Okazawa et al., 2012), a marker of 3D convexity for certain materials, and TEs, a region extracting curvature from disparity (Janssen et al., 2000). This substream overlaps with action-processing regions located in both banks of the STS, especially their deeper regions (Nelissen et al., 2011). One of the main cues for extracting actions is the deformation of body shape (Vangeneugden et al., 2009; Singer and Sheinberg, 2010), explaining the proximity of shape, and action processing areas. Similarly, material properties contribute heavily to scene processing, which may explain their location in ventral TE, as it neighbors the scene-processing stream in parahippocampal TF/TH.

Both the general anatomy, that indicates serial processing (**Figure 2A**), and studies specific to the face-processing system suggest that the representation of RWEs might be further elaborated rostrally within TE. A detailed study of the face patches (Freiwald and Tsao, 2010) suggests that the first step is the extraction of the face category in ML; that additional properties, such as the viewpoint from which the face is seen, are represented in subsequent patches; and that finally at the highest level, exemplars, individual faces, are represented, implying that sufficient invariance has been achieved. Similarly Lafer-Sousa and Conway (2013) have suggested that the representation of color is more elaborated in anterior than in posterior TE. Koida and Komatsu (2007) demonstrated the task dependent activity of TE color selective neurons. Task dependent processing and other aspects of TE processing such as extending the neural representation beyond the stimulus presentation (Kovacs et al., 1995) or buffering the last representation (Orban and Vogels, 1998) are beyond the scope of the present perspective paper.

Despite this elaboration of RWE representations, including becoming gradually more invariant (DiCarlo et al., 2012), the representation in TE remains incomplete in the sense that the entire RWE is generally not represented (a few neurons may do so, as suggested for target-paired association neurons; Hirabayashi et al., 2013). Even in the anterior face patches, only the face is represented, not the whole person; also, patches related to color represent only one material aspect of the RWE. The partial representation of the RWE at the middle level can be considered a generalization of the selectivity of TE neurons for 2D shape components (Tanaka et al., 1991). The RFs of TE neurons are relatively large (about 15^∘^ diameter), located primarily in the contralateral visual field, and generally included the fovea (Op De Beeck and Vogels, 2000). Hence a certain spatial coding remains possible, in particular that of the relative positions of shape or RWE parts. Several rationales can be advanced for the incomplete representation of RWEs in TE having to do with more flexible representations. In particular, some material properties define the exemplar but not the category (e.g., John may have black hair but not all men have black hair), accommodation of slow changes in properties, e.g., due to aging, or seasons (color changes of the leaves), and finally detection of uncommon associations of shape and color (see Zeki and Marini, 1998; e.g., John generally looks healthy, but can be very pale because of illness).

Thus far, views about the organization of TE have been dominated by the presence of patches in TE, among which face and body patches (Tsao et al., 2003; Pinsk et al., 2009; Bell et al., 2011; Popivanov et al., 2012) are the best known. Initially it was assumed that the non-face and non-body objects were processed outside these patches (Ishai et al., 1999; Tsao et al., 2003), implying that RWE of different types were processed in different compartments of TE. This view, however, is inconsistent with recent evidence for patches for color, 3D shape from disparity, or gloss (Harada et al., 2009; Joly et al., 2009; Okazawa et al., 2012). A recent study by Srihasam et al. (2012) sheds new light on the exact organization of TE. These authors showed that when monkeys are trained to use numerical or letter symbols from a young age, these stimuli are represented in patches within TE, but are not present in untrained monkeys or those trained to use these symbols as adults (and not learning the task as well). While others (Vogels and Orban, 1994; Kobatake et al., 1998; Sigala and Logothetis, 2002) have reported plasticity at the single-cell level after training, the Srihasam study was the first to report functional architectural changes in TE, rather than just changes in neuronal properties. Srihasam et al. (2012) suggest that patches arise because neurons with similar selectivity tend to group together to increase computational efficiency (shorter connections). In retinotopic cortex, these groupings are constrained by the retinotopic organization, but in TE this is not the case, thus giving rise to varying degrees of aggregation, probably depending on the behavioral relevance of the selectivity. Those aspects or components of RWEs with strong behavioral relevance are grouped into complex systems of multiple connected patches, of which the face patches are probably the most elaborated. Those with limited relevance, such as properties/parts of objects encountered only infrequently, have small representations in columnar-like structures (Tanaka et al., 1991). Those with intermediate relevance have a somewhat broader representation, in one or two patches, such as color or 3D shape. Thus the processing of RWEs of different type or nature is interwoven, their properties being represented more or less extensively depending on behavioral relevance. Such size differences of functional TE modules are consistent with the findings of Sato et al. (2013), with our largest and smallest modules corresponding to their domains and columns, respectively. In humans these domains may include the word form areas (Cohen et al., 2000) analyzing strings of symbols during reading, even if words are not actually RWEs.

## REPRESENTATIONS OF ACTIONS IN STS

Several lines of investigation suggest that actions (purposeful movements of an agent: animal, human, or even robot) are processed in the middle and rostral STS largely in parallel with RWEs in TE (**Figure 3**). Recent evidence suggests that actions are extracted in LST and STPm, two motion-sensitive regions just anterior to the MT cluster. In these regions the configuration and kinematic cues of BM interact (Jastorff et al., 2012), which is the definition of action. Indeed, action-selective neurons have been recorded at this level, and both cues appear operative: deforming shape in the lower bank, and motion patterns in the upper bank (Vangeneugden et al., 2009). We have begun to understand the homology of monkey STS (Orban and Jastorff, 2014): The lower bank corresponds to posterior OTS and fusiform cortex in humans, overlapping with LOC (in which actions and shape overlap, as in the lower bank of STS; Jastorff and Orban, 2009), while the upper bank of monkey STS corresponds to posterior MTG and posterior STS in humans (Jastorff and Orban, 2009; Jastorff et al., 2012).

We have recently shown that the action-sensitive regions of STS devoted to grasping project to the ventral premotor cortex (F5), where mirror neurons occur, via two way stations in the PPC: AIP and PFG (Nelissen et al., 2011). We believe that this is a general strategy within the primate visual system, not merely for grasping and manipulative actions, but for all types of action. The STS action-processing regions project to the PPC in order to extract action category which requires that a large number of invariances to be solved: not only for size, position, and in plane orientation, as for RWEs, but also for viewpoint and posture. The available evidence (Freiwald and Tsao, 2010) suggests that TE and neighboring regions achieve invariance only at the expense of large neuronal pools and that therefore the many invariances required for understanding body actions involve too much neuronal hardware to be realistically achieved in the STS. Hence, we propose that the STS regions send the visual information about *which action* is observed to the PPC housing the schema of specific actions, i.e., the sensori-motor transformation underlying various actions. By projecting these visual signals onto the corresponding motor plan, invariance is automatically achieved and categorization becomes feasible. This invariance problem is less stringent for facial expressions, as the viewpoints, and postural invariance requirements are much more limited. Hence what applies to body action may not necessarily apply to facial expressions, explaining the presence of face patches in the upper bank of STS, where dynamic face expressions are processed (Polosecki et al., 2013).

These action signals sent to the PPC concern the nature/goal of the action defining *which action* is observed. However, actions are also further processed in the STS itself, analysis probably related to *how the action* is performed, e.g., slowly or quickly, with difficulty or easily, physiologically or pathologically. The latter sort of processing provides information about the state of the actor, even if the actor itself, an RWE, is processed in TE. The state of the agent reflects his/her emotions, but also the physiological state, and perhaps also vitality (Di Cesare et al., 2013). The latter aspect is related to the rank of the actor in the group or the social organization in general and may be dealt with in human TPJ, a region which may have arisen from some middle part of the STS (Sallet et al., 2011; Mars et al., 2013). TPJ is often considered the starting point (Saxe et al., 2004) for processing other agents (theory of mind), but recent studies (Jastorff and Orban, 2009) alternatively suggest that there might be a representation of an agent in the scene in posterior STG. Activity in posterior STS and TPJ would then specify properties of the agent, such as rational or efficient behavior (Jastorff et al., 2011).

## THREE LEVELS OF PROCESSING IN THE VENTRAL STREAM (FIGURE 3)

TE corresponds to the middle level of the ventral stream in the monkey. It builds a partial representation of RWEs and operates in parallel with STS, processing actions and TF/TH processing scenes (**Figure 3**). TE receives input from retinotopic cortex (first level) where image features are processed to generate higher-order features related to 3D shape, 2D shape, or material properties in specific parts of the visual field. The retinotopic visual cortex not only processes a range of elementary image features (Zeki, 1978) but also resolves image segmentation by establishing topological relationships between the features: inside vs. outside and in front vs. behind (Zhang and von der Heydt, 2010). The anatomy indicates, however, that the ventral pathway in monkeys may include, in addition to the retinotopic cortex and TE, a third level beyond TE. A small temporal region, TGv, receiving input from the three substreams in TE, is situated in front of TE near the temporal pole (Kravitz et al., 2013). The TGv region projects to rhinal cortex in which memory of the association between two images is constructed by the convergence of their representations in TE (Naya et al., 2003a; Hirabayashi et al., 2013). We propose that the TGv region, which is greatly expanded in humans and is referred to as the temporal pole, builds on the partial representations of individual RWEs achieved at the rostral TE (Freiwald and Tsao, 2010) to generate representations of *known* RWEs (Damasio et al., 2004; Quiroga et al., 2005). The association of the elements present in TE detected in rhinal cortex (Hirabayashi et al., 2013), may be back-projected (Naya et al., 2003b; Takeuchi et al., 2011) onto the most rostral visual part of temporal cortex, giving rise to representations of known RWEs (Takeda et al., 2005). For example, exemplars of a shape category, e.g., face plus body, and particular material properties define a given individual and this association gives rise to the representation of that known individual in TGv, perhaps supplemented by information about *how* he acts and the scenes in which he appears. In contrast to the TE level, the representation here is that of the complete RWE, e.g., a conspecific, and no longer simply a face. A similar operation may be applied to scene information in parahippocampal areas, giving rise to known places, although no direct link between TF/TH and TGv has been described. Interestingly, recent fMRI data (Miyamoto et al., 2014) indicate that monkey rhinal cortex encodes familiar items, operationalized as middle items in a serial probe task. This type of encoding is appropriate for known RWEs, and by extension, semantic knowledge. In humans, this third level of the ventral stream, the temporal pole, may correspond to the anterior part of the semantic system (Vandenberghe et al., 1996). This association between the temporal pole and semantic memory has its basis in the connections of the pole to memory structures such as rhinal cortex. The third level may also be linked with the amygdala, the structure underlying association between known person and emotions, which has been referred to as personal semantic memory (Olson et al., 2007).

The visual representation of known RWEs at the third level also seems consistent with single cell recorded in the human hippocampal complex showing neuronal selectivity for familiar persons or places, sometimes referred to as visual concept neurons (Quiroga, 2012). This might suggest that visual episodes (events) are also represented at this third level and probably beyond, e.g., in entorhinal cortex and hippocampus. The latter view is supported by the recent study of Miyamoto et al. (2014), who showed that the memory trace of recalled items, operationalized as the first item in a serial probe task, is located in caudal entorhinal cortex and hippocampus of the monkey. A relatively small region may suffice for representing episodes, as this representation may be short-lived. Indeed, if the event is repeated or memorable it may become knowledge (the fact that somebody looks ill may become part of medicine or history); if it is important for the subject it may become part of autobiographic memory. The dissociation of episodic and semantic memory within the third, known-RWE level is also supported by patients studies (Hirni et al., 2013).

For simplicity we have described the three levels, those processing features, partial RWEs, and known RWEs, as separate components, using anatomy (Kravitz et al., 2013) as a guide. It is possible, however, that the transitions between these levels are gradual. Indeed, as mentioned, the ML face patch is located at the edge of retinotopic cortex and the overlap between retinotopic cortex and some of the more caudal face or body patches may be larger in humans than in monkeys. In the monkey, the body patch is anterior to the MT cluster (Jastorff et al., 2012), but in humans EBA overlaps the retinotopic MT cluster to a large extent (Ferri et al., 2012). Moreover, segregation between the third level, TGv, and the levels below, TE, and beyond, rhinal cortex, might be incomplete, insofar as the anterior parts of TE and the lower bank of STS also exchange bidirectional projections with rhinal cortex. At this level, differences between humans and monkeys may have arisen due to the enlargement of the temporal pole in humans.

The three levels of the ventral stream also appear to differ in the way they develop. The experiment of Srihasam et al. (2012) suggests that the middle level (TE, and by extension perhaps also STS and TF/TH) reflects the individual development, while the earlier retinotopic level is probably species-specific. This explains that although the different retinotopic regions are present in all individual subjects, albeit with some variation in size and location, the number of patches in TE seems more variable among individuals (Bell et al., 2011; Lafer-Sousa and Conway, 2013). The third and final level would remain the most plastic and dependent on lifelong mental activity. Its internal organization is presently unknown.

In *conclusion* we propose that the ventral stream is organized into three levels comprising the ventral retinotopic cortex known as TEO, TE, and TGv in the monkey, and their homologs in human cortex. We attribute to these levels the visual representation of features, partial RWEs, and more speculatively, known, complete RWEs, respectively. Furthermore, the middle level TE and its human equivalent is organized into three parallel substreams related to processing shape in depth, 2D shape, and material properties in order to build up RWE representations.

## Conflict of Interest Statement

The authors declare that the research was conducted in the absence of any commercial or financial relationships that could be construed as a potential conflict of interest.

## Abbreviations

**Cortical areas and regions**
AIP: anterior intraparietal area
CIP: caudal intraparietal area
DP: dorsal parietal area, located dorsal from V4
FST: fundus of superior temporal area, third element of the MT, cluster
pFST: human homolog of FST
IPS0-5: is a set of successive retinotopic areas near the intraparietal sulcus (IPS) defined solely by reversal of polar angle (visual field is defined by both polar angle and eccentricity); IPS0-1 corresponds to V7/V7A
IT: infero-temporal cortex, includes three cytoarchitectonic fields, TEO, TE, and TGv; the first two have also been parceled into three antero-posterior subdivisions, posterior IT (PIT), central IT (CIT) and anterior IT (AIT), with PIT largely corresponding to TEO and CIT and AIT to TE; It includes the lower bank of the superior temporal sulcus (STS)
LO1, LO2: lateral occipital area 1 and 2
LOC: lateral occipital cortex defined by the contrast *intact* vs *scrambled images* of objects. Includes LO1-2 but extends rostrally into occipito-temporal sulcus and fusiform cortex
LST: lateral superior temporal area, a motion area located in the monkey STS in front of FST
MSTv: medial superior temporal area ventral part, second component of the MT cluster; pMSTv human homologue of MSTv
MT: middle temporal area; first element of the MT cluster
OTd: occipito-temporal dorsal area
PFG: cytoarchitectonic field in IPL (others are PF, PG, and opt)
PITd: posterior infero-temporal dorsal area; phPITd, putative human homologue of PITd
PITv: posterior infero-temporal ventral area; phPITv, putative human homologue of PITv
PPC: posterior parietal cortex (part of parietal cortex behind primary somato-sensory cortex)
STPm: superior temporal posterior middle area, a motion area located in the upper bank of monkey STS (middle level)
TF, TH: cytoarchtectonic regions of parahippocampal cortex
TFO: cytoarchitectonic area posterior to TF/TH and medial to TEO; has been labeled previously VTF (visual part of TF) by Boussaoud et al. (1991), but is now recognized as a separate cytoarchitectonic entity (Kravitz et al., 2013)
V1, V2-V7: visual area 1, 2, to 7. The designation “V7” has been used only in humans; V5 corresponds to MT; While homology for V1-3 and V5/MT and V6 is relatively well established, hV4 refers a human area in positioned similarly to monkey V4 but having a different retinotopic organization; V3A, V4A, ad V7A, areas in neighborhood of V3, V4, and V7
V4t: fourth area of the MT cluster, initially considered incomplete now, accepted as corresponding to a complete hemifield; pV4t, human homologue of V4t
VO1, VO2: ventral occipital area 1 and 2

**Anatomical structures**
IPS: intraparietal sulcus separating the superior parietal lobule (SPL) from the inferior parietal lobule (IPL)
MTG: middle temporal gyrus
OTS: occipito-temporal sulcus
STS: superior temporal sulcus
STG: superior temporal gyrus
TPJ: temporo-parietal junction

**Other abbreviations:**
AL: anterior lateral (face patch)
BM: biological motion
ML: middle lateral (face patch)
RWE: real world entity.
